# Fasting hepatic glucose uptake is higher in men than women

**DOI:** 10.14814/phy2.13174

**Published:** 2017-06-05

**Authors:** Georgia Keramida, A. Michael Peters

**Affiliations:** ^1^ Clinical Imaging Sciences CentreBrighton and Sussex Medical SchoolBrightonUnited Kingdom; ^2^ Division of Clinical and Laboratory InvestigationBrighton and Sussex Medical SchoolBrightonUnited Kingdom

**Keywords:** FDG, gender, glucose, liver, PET/CT

## Abstract

Differences in glucose metabolism between men and women have previously been reported. Our purpose was to determine if there is a gender difference in fasting hepatic glucose uptake (MRglu). Fifty‐five patients (44 men, 11 women) referred for routine PET/CT using the glucose tracer 2‐deoxy‐2‐[F‐18]fluoro‐D‐glucose (FDG), mainly for cancer, had dynamic imaging for 30 min immediately following injection. Hepatic FDG clearance (mL/min/100 mL) was measured as gradient divided by intercept from Patlak–Rutland graphical analysis using a volume of interest over the abdominal aorta to record input function. Hepatic MRglu was obtained by multiplication of clearance by blood glucose concentration. Hepatic steatosis was diagnosed as CT density ≤40 HU. Mean (standard deviation) hepatic MRglu in 44 men was 2.30 (1.14) *μ*mol/min/100 mL, significantly higher than in 11 women in whom it was 1.07 (1.35) *μ*mol/min/100 mL (*P* = 0.003). CT density was 52 (12) HU in women compared with 45 (9) HU in men (*P* = 0.04), but there was no significant difference in blood glucose, BMI, or prevalence of recent chemotherapy (within 6 months preceding PET/CT). When patients were subdivided into those without hepatic steatosis (31 men/9 women), those without evidence of FDG‐avid malignancy on PET/CT (15/6), and those without either (11/5), gender differences in hepatic MRglu remained highly significant, but there were no significant differences in CT density, blood glucose, BMI, or recent chemotherapy history. Despite this being a population of clinically referred patients, the results strongly suggest that fasting hepatic MRglu is higher in men than in women.

## Introduction

There are differences between men and women with respect to glucose metabolism (Basu et al. 2006; Horton et al. 2006; Blaak 2008; Magkos et al. 2010; Varlamov et al. 2015). For example, compared with men, women are less glucose tolerant (Blaak 2008), have a lower hepatic extraction fraction of insulin (Basu et al. 2006), and produce less glucose in response to exercise (Horton et al. 2006). Women and the females of other species are also more susceptible to the effects of hepatotoxins, such as alcohol (Tuyns and Pequignot 1984; Iimuro et al. 1997), and there are gender differences in the natural history of liver disease (Guy and Peters 2013). However, data on gender differences in fasting hepatic glucose uptake (MRglu) are more difficult to find.

**Figure 1:**
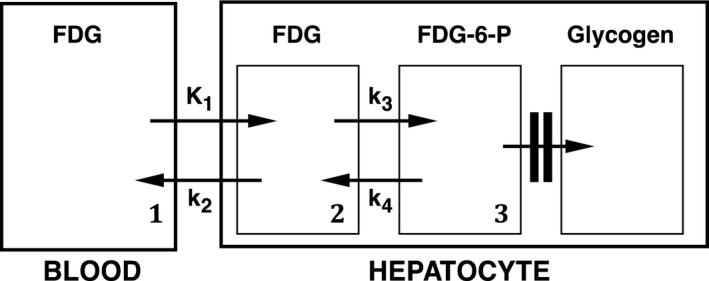
Model of hepatic FDG kinetics. K_1_ is hepatic blood flow, k_2_ is a diffusion constant, k_3_ is hexokinase, and k_4_ is glucose‐6‐phosphatase. FDG is assumed to mix throughout its intrahepatic distribution volume (compartments 1 and 2) via K_1_ and k_2_ by 2 min post injection. Dephosphorylation (via k_4_) in compartment 3 is assumed to be slow enough to ignore. Patlak–Rutland analysis therefore measures k_3_. FDG, 2‐deoxy‐2‐[F‐18]fluoro‐D‐glucose.

Previous studies have used the glucose tracer, 2‐deoxy‐2‐[F‐18]fluoro‐D‐glucose (FDG) and positron emission tomography (PET) to measure hepatic MRglu (Choi et al. 1994; Iozzo et al. 2003; Borra et al. 2008), but only in men. Like glucose, FDG freely exchanges between blood and hepatocytes via K_1_ (close to hepatic perfusion) and k_2_ (diffusion constant) (Choi et al. 1994; Torizuka et al. 1995; Green et al. 1998) (Fig 1). In the hepatocyte, FDG is phosphorylated to FDG‐6‐phosphate via hexokinase (transport pathway k_3_). Dephosphorylation through glucose‐6‐phosphatase (k_4_) appears to be relatively slow compared with phosphorylation, even in fasting patients (Choi et al. 1994; Iozzo et al. 2003), so that dynamic imaging over a limited period of time following FDG injection with graphical analysis of data can be used to measure hepatic FDG clearance (Choi et al. 1994; Munk et al. 2001; Iozzo et al. 2003).

The aim of the study was to determine if there is a difference in hepatic MRglu between men and women.

## Methods

### Compliance with ethical standards

Georgia Keramida and Adrien Michael Peters declare that they have no conflict of interest to declare.

### Ethical approval

All procedures performed in studies involving human participants were in accordance with the ethical standards of the institutional and/or national research committee and with the 1964 Helsinki declaration and its later amendments or comparable ethical standards.

### Patients

Sixty patients referred for clinically indicated PET/CT (57 for management of cancer and three for unexplained fever) were recruited. No specific selection criteria were used other than exclusion of patients with suspected high alcohol intake (confessing to >21 units per week). These patients also formed the population in a previous study that investigated the relationships of hepatic MRglu with hepatic steatosis and obesity, published elsewhere (Keramida et al. 2016). As previously described, they had dynamic PET for 30 min following FDG injection (~400 MBq) in a single bed position with detector over the torso. This was followed by routine whole‐body PET/CT (Siemens Healthcare, Erlangen, Germany) at 60 min post injection, again as previously described (Keramida et al. 2016).

Three men and 2 women with type‐2 diabetes mellitus were excluded, leaving 44 men (mean age 61 ± standard deviation [SD] 12) and 11 women (mean age 60 ± 10). Thirteen men and 2 women had hepatic steatosis, defined from the CT component of PET/CT as CT density of ≤40 HU (Boyce et al. 2010). PET/CT imaging revealed no FDG‐avid pathology (“metabolically active” disease) in 15 men and six women. Hepatic MRglu was compared between men and women in all 55 patients and three subgroups: firstly, in 40 without hepatic steatosis; secondly, in 21 without PET/CT evidence of metabolically active disease; and thirdly, in 16 without hepatic steatosis or evidence of metabolically active disease (Table 1). Of the 44 men, 11 had received chemotherapy within the 6 months preceding their PET/CT scan, while the corresponding proportion for women was 1/11 (*P *=* *0.12).

**Table 1:** Mean (standard deviation) hepatic glucose uptake (MRglu), CT density, blood glucose concentration, body mass index (BMI), and recent chemotherapy history compared between men and women in 55 patients and three subgroups

	MRglu* μ*mol/min/100 mL	CT Density HU	Blood glucose mmol/L	BMI kg/m^2^	Chemotherapy within 6 months
All patients					
Men (*n* = 44)	2.30 (1.14)	45 (9)	5.7 (0.6)	27 (5)	11/44
Women (*n* = 11)	1.07 (1.35)	52 (12)	5.8 (0.7)	26 (6)	1/11
*P*	0.003	0.04	NS	NS	NS
No steatosis					
Men (*n* = 31)	2.04 (1.11)	50 (4)	5.7 (0.6)	25 (4)	6/31
Women (*n* = 9)	0.64 (0.76)	56 (7)	5.6 (0.5)	25 (5)	1/9
*P*	0.0009	NS	NS	NS	NS
No FDG‐avid malignancy					
Men (*n* = 15)	2.41 (0.99)	46 (11)	5.7 (0.6)	26 (5)	4/15
Women (*n* = 6)	0.54 (0.66)	50 (9)	5.6 (0.6)	28 (3)	1/6
*P*	0.0004	NS	NS	NS	NS
No steatosis or FDG‐avid malignancy					
Men (*n* = 11)	2.21 (1.03)	52 (5)	5.6 (0.5)	24 (2)	2/11
Women (*n* = 5)	0.34 (0.48)	53 (6)	5.5 (0.6)	28 (4)	1/5
*P*	0.002	NS	NS	NS	NS

*P* refers to the difference between men and women (Student unpaired *t*‐test). NS, statistically insignificant (*P *>* *0.05).

### Dynamic imaging

**Figure 2:**
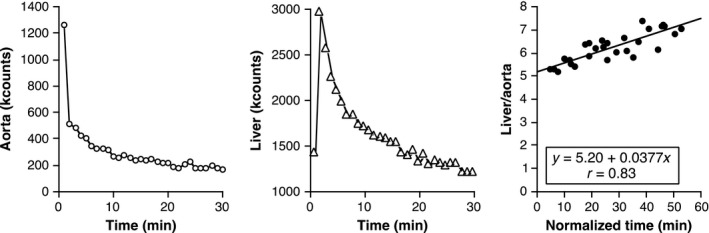
Example of time‐count curves for the aortic blood pool (left panel), liver (middle panel) (kcounts = counts/frame in thousands), and Patlak–Rutland plot based on these curves (right panel). Note that the first two frames are not included in the Patlak–Rutland plot.

Hepatic FDG clearance was measured from dynamic imaging using Patlak–Rutland (PR) graphical analysis (Choi et al. 1994; Munk et al. 2001; Iozzo et al. 2003; Borra et al. 2008) and *Hermes* software (Hermes Medical Solutions, Stockholm, Sweden), as previously described (Keramida et al. 2016). Hepatic activity was summed from regions of interest of 3.0 cm diameter over the right lobe on about contiguous 20 transaxial images, avoiding any suspected focal hepatic pathology in each transaxial section. Blood pool activity was obtained similarly from ROIs of 1.6 cm diameter on about 20 transaxial images of the abdominal aorta within the field of view, being careful to place the ROI within the lumen, which, on early imaging, is conspicuous. The ratio of hepatic‐to‐aortic counts was then plotted against the ratio of integral of aortic counts‐to‐aortic counts (Fig 2). The latter has units of time (‘normalized time”). The gradient of the PR plot is proportional to hepatic FDG clearance (Ki) and the intercept is proportional to the distribution volume (V(0)) of unphosphorylated FDG throughout the liver. The proportionality constants (based on the relation of tissue counts and blood counts to tissue and blood concentrations FDG) are identical, as proved elsewhere (Keramida et al. 2016); so, Ki was divided by V(0) to give hepatic FDG clearance per unit FDG hepatic distribution volume. This requires no attenuation correction because the proportionality constants cancel out. As FDG presumably does not penetrate hepatic fat deposits, this approach has the advantage that the physical impact of hepatic fat on the FDG signal (Keramida et al. 2014) also cancels out because it affects gradient and intercept equally. Ki/V(0) was then multiplied by blood glucose to give hepatic MRglu (*μ*mol/min/ml). Lumped constant (LC) for the liver was assumed to be unity (Iozzo et al. 2007).

### Statistics

Results are expressed as mean ± standard deviation. Differences between men and women were evaluated using Student's unpaired *t*‐test. Correlations were based on Pearson correlation analysis. Differences were considered nonsignificant when *P* > 0.05.

### Ethics

All patient data are anonymous. All patients attending our PET/CT unit are asked for permission to use any of their imaging data for research purposes (a practice approved by a National Research Ethics Committee) and all patients consented. Moreover, the acquisition of the dynamic data (which did not require any additional radiation exposure and for which all patients gave written informed consent) was approved by a National Research Ethics Committee.

## Results

**Figure 3:**
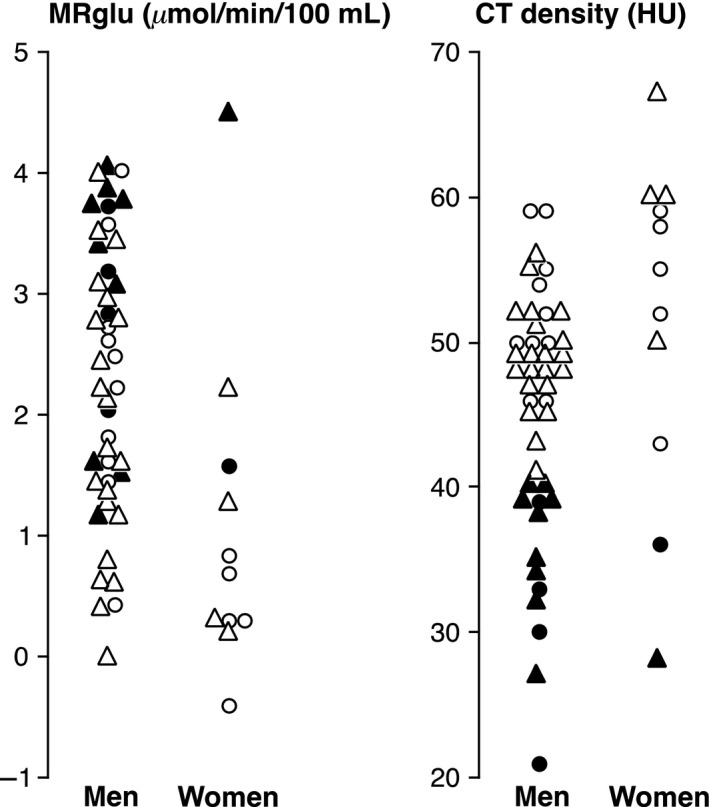
Hepatic MRglu (left panel) and CT density (right panel) in men versus women. Open symbols: patients with no hepatic steatosis; closed symbols: patients with hepatic steatosis; triangles: patients with FDG‐avid pathology on routine PET/CT; circles: patients without FDG‐avid pathology.

Hepatic MRglu was approximately twice as high in men (2.30 ± 1.14 *μ*moL/min/100 mL) compared with women (1.07 ± 1.35 *μ*mol/min/100 mL; *P* = 0.003) in all 55 patients (Fig 3; Table 1). The difference was equally marked in the 40 with no steatosis, the 21 with normal PET/CT and the 16 with no steatosis and normal PET/CT (Fig 3; Table 1). There were no significant differences in blood glucose, body mass index (BMI), or recent chemotherapy history in any of the subgroups. CT density, however, was minimally but significantly higher in women when all 55 patients were considered but not in the subgroups (Table 1). When patients with hepatic steatosis were excluded, the gender difference in hepatic MRglu was still marked. There was no correlation between hepatic MRglu and age in either gender. Mean hepatic MRglu in 11 men with a history of chemotherapy within 6 months of their PECT/CT scan was 2.54 ± 0.90 *μ*mol/min/100 mL compared with 2.22 ± 1.21 *μ*mol/min/100 mL in 33 men who had not received chemotherapy within 6 months (*P* > 0.05).

## Discussion

We show in a heterogeneous clinical population that there is a marked difference in hepatic MRglu between men and women that cannot be explained by differences in age, BMI, hepatic CT density, or recent chemotherapy administration. This complements previous work highlighting differences between men and women in glucose and fat metabolism, as described above.

The mechanism for this gender‐specific difference in hepatic MRglu is not apparent, although sex hormones may have a role (Shen and Shi 2015). The phosphorylation transport constant, k_3_, is sensitive to insulin and was shown by Iozzo et al. (2003) to remain sensitive to insulin in patients with low insulin sensitivity. Insulin resistance, which is more common in hepatic steatosis (Marchesini et al. 1999), may play a role, except the gender difference in hepatic MRglu was still seen when 15 patients with hepatic steatosis were excluded. Nevertheless, as hepatic MRglu was measured under fasting conditions, during which insulin levels would have been low, an insulin‐mediated mechanism seems unlikely.

Previous studies (Choi et al. 1994; Munk et al. 2001; Iozzo et al. 2003) used kinetic modeling to determine transport rates of FDG between hepatocytes and blood (K_1_ and k_2_), and rate constants of FDG phosphorylation (k_3_) and dephosphorylation (k_4_). Modeling is complicated by the liver's dual blood supply (Munk et al. 2001; Tragardh et al. 2015) but PR analysis, although yielding only Ki/V(0) rather than individual transfer constants, is more robust for measuring hepatic MRglu (Choi et al. 1994; Munk et al. 2001; Iozzo et al. 2003). We, like others (Choi et al. 1994; Munk et al. 2001; Iozzo et al. 2003), found the hepatic PR plot to be essentially linear, suggesting that k_4_ makes no significant impact. Nevertheless, we limited our acquisition period to 30 min not only to minimize the potential effects of k_4_ but also to minimize patient movement artifact, as unsedated patients find it difficult to lie still for more than 30 min. Our assumption of completion of mixing of FDG within its hepatic distribution volume within 3 min appears valid from the linearity of the PR plot from 3 min (Fig 1).

Our values of hepatic MRglu in men are very similar to values previously recorded by others who measured blood and liver FDG concentrations in absolute units (MBq/ml) rather than as raw counts (Choi et al. 1994; Iozzo et al. 2003; Borra et al. 2008). Thus, in healthy men, Choi et al. (1994) recorded a fasting hepatic MRglu of 2.1 *μ*mol/min/100 mL, where 100 mL refers to total liver volume rather than V(0). The Finnish group obtained fasting values of 1.3 (Iozzo et al. 2003) and 3.6 (Borra et al. 2008) *μ*mol/min/100 mL, again, in men. Choi et al. found V(0) to be 0.88 mL/mL. It is likely to be lower in hepatic steatosis. Division of their mean hepatic MRglu by 0.88 gives 2.4 *μ*mol/min/100 mL, which is the value that should be compared with ours.

FDG presumably does not penetrate the fat droplets within hepatocytes; so, overall hepatic FDG concentration will be reduced by the presence of hepatic fat (Keramida et al. 2014), thereby reducing the value of Ki. However, V(0) will be reduced equally, so dividing Ki by V(0) avoids this signal dilution effect, which could be prominent in severe hepatic steatosis.

Study imitations are as follows. Firstly, it would have been informative to measure blood insulin levels, as insulin stimulates k_3_ and increases hepatic MRglu (Choi et al. 1994; Iozzo et al. 2003). In this respect, similar measurements made during glucose challenge, such as performed by Choi et al. (1994), or hyperinsulinaemic euglycaemic clamp, such as performed by Iozzo et al. (2003), may help identify gender differences related to the role of insulin in mediating hepatic glucose uptake. Secondly, we assumed LC of unity for both genders, although it is unlikely that LC would differ between men and women. Thirdly, our patient population was clinically heterogeneous rather than a healthy one and included patients with hepatic steatosis. Previous studies on the relation between hepatic steatosis and hepatic MRglu are few and conflicting. However, we still saw a rather striking difference between hepatic MRglu in men and women when patients with hepatic steatosis and/or FDG‐avid pathology were excluded. Finally, excluding patients with excessive alcohol intake may have been incomplete.

In conclusion, we present evidence that fasting hepatic MRglu is higher in men than in women. This may have implications for differences in susceptibility that exist between men and women with respect to liver disease. Further study of healthy men and women, matched for age, BMI, and CT density is warranted for confirmation of this observation.

## Conflict of Interest

None declared.

## References

[phy213174-bib-0001] Basu, R. , C. Dalla Man , M. Campioni , A. Basu , G. Klee , G. Toffolo , et al. 2006 Effects of age and sex on postprandial glucose metabolism differences in glucose turnover, insulin secretion, insulin action, and hepatic insulin extraction. Diabetes 55:2001–2014. 16804069 10.2337/db05-1692

[phy213174-bib-0002] Blaak, E. 2008 Sex differences in the control of glucose homeostasis. Curr. Opin. Clin. Nutr. Metab. Care 11:500–504. 18542013 10.1097/MCO.0b013e32830467d3

[phy213174-bib-0003] Borra, R. , R. Lautamaki , R. Parkkola , M. Komu , P. E. Sijens , K. Hällsten , et al. 2008 Inverse association between liver fat content and hepatic glucose uptake in patients with type 2 diabetes mellitus. Metabolism 57:1445–1451. 18803951 10.1016/j.metabol.2008.05.015

[phy213174-bib-0004] Boyce, C. J. , P. J. Pickhardt , D. H. Kim , A. J. Taylor , T. C. Winter , R. J. Bruce , et al. 2010 Hepatic steatosis (fatty liver disease) in asymptomatic adults identified by unenhanced low‐dose CT. Am. J. Roentgenol. 194:623–628. 20173137 10.2214/AJR.09.2590

[phy213174-bib-0005] Choi, Y. , R. A. Hawkins , S. C. Huang , R. C. Brunken , C. K. Hoh , C. Messa , et al. 1994 Evaluation of the effect of glucose ingestion and kinetic model configurations of FDG in the normal liver. J. Nucl. Med. 35:818–823. 8176464

[phy213174-bib-0006] Green, L. A. , S. S. Gambhir , A. Srinivasan , P. K. Banerjee , C. K. Hoh , S. R. Cherry , et al. 1998 Noninvasive methods for quantitating blood time‐activity curves from mouse PET images obtained with fluorine‐18‐fluorodeoxyglucose. J. Nucl. Med. 39:729–734. 9544690

[phy213174-bib-0007] Guy, J. , and M. G. Peters . 2013 Liver disease in women: the influence of gender on epidemiology, natural history, and patient outcomes. Gastroenterol. Hepatol. 9:633–639. PMC3992057 24764777

[phy213174-bib-0008] Horton, T. J. , G. K. Grunwald , J. Lavely , and W. T. Donahoo . 2006 Glucose kinetics differ between women and men, during and after exercise. J. Appl. Physiol. 100:1883–1894. 16714415 10.1152/japplphysiol.01431.2005

[phy213174-bib-0009] Iimuro, Y. , M. V. Frankenberg , G. E. Arteel , B. U. Bradford , C. A. Wall , and R. G. Thurman . 1997 Female rats exhibit greater susceptibility to early alcohol‐induced liver injury than males. Am. J. Physiol. 272:G1186–G1194. 9176229 10.1152/ajpgi.1997.272.5.G1186

[phy213174-bib-0010] Iozzo, P. , F. Geisler , V. Oikonen , M. Mäki , T. Takala , O. Solin , et al. 2003 Insulin stimulates liver glucose uptake in humans: an ^18^F‐FDG PET Study. J. Nucl. Med. 44:682–689. 12732668

[phy213174-bib-0011] Iozzo, P. , M. J. Jarvisalo , J. Kiss , R. Borra , G. A. Naum , A. Viljanen , et al. 2007 Quantification of liver glucose metabolism by positron emission tomography: validation study in pigs. Gastroenterology 132:531–542. 17258736 10.1053/j.gastro.2006.12.040

[phy213174-bib-0012] Keramida, G. , J. Potts , J. Bush , S. Verma , S. Dizdarevic , and A. M. Peters . 2014 Accumulation of ^18^F‐FDG in the liver in hepatic steatosis. Am. J. Roentgenol. 203:643–648. 25148170 10.2214/AJR.13.12147

[phy213174-bib-0013] Keramida, G. , J. Hunter , and A. M. Peters . 2016 Hepatic glucose utilisation in hepatic steatosis and obesity. Biosci. Rep. pii: BSR20160381. 10.1042/BSR20160381 PMC5293565 27653524

[phy213174-bib-0014] Magkos, F. , X. Wang , and B. Mittendorfer . 2010 Metabolic actions of insulin in men and women. Nutrition 26:686–693. 20392600 10.1016/j.nut.2009.10.013 PMC2893237

[phy213174-bib-0015] Marchesini, G. , M. Brizi , A. M. Morselli‐Labate , G. Bianchi , E. Bugianesi , A. J. McCullough , et al. 1999 Association of nonalcoholic fatty liver disease with insulin resistance. Am. J. Med. 107:450–455. 10569299 10.1016/s0002-9343(99)00271-5

[phy213174-bib-0016] Munk, O. L. , L. Bass , K. Roelsgaard , D. Bender , S. B. Hansen , and S. Keiding . 2001 Liver kinetics of glucose analogs measured in pigs by PET: importance of dual‐input blood sampling. J. Nucl. Med. 42:795–801. 11337579

[phy213174-bib-0017] Shen, M. , and H. Shi . 2015 Sex hormones and their receptors regulate liver energy homeostasis. Int. J. Endocrinol 2015:294278. doi:10.1155/2015/294278. 26491440 10.1155/2015/294278 PMC4600502

[phy213174-bib-0018] Torizuka, T. , N. Tamaki , T. Inokuma , Y. Magata , S. Sasayama , Y. Yonekura , et al. 1995 In vivo assessment of glucose metabolism in hepatocellular carcinoma with FDG PET. J. Nucl. Med. 36:1811–1817. 7562048

[phy213174-bib-0019] Tragardh, M. , N. Moller , and M. Sorensen . 2015 Methodologic considerations for quantitative ^18^F‐FDGPET/CT studies of hepatic glucose metabolismin healthy subjects. J. Nucl. Med. 56:1366–1371. 26159590 10.2967/jnumed.115.154211

[phy213174-bib-0020] Tuyns, A. J. , and G. Pequignot . 1984 Greater risk of ascitic cirrhosis in females in relation to alcohol consumption. Int. J. Epidemiol. 13:53–57. 6698704 10.1093/ije/13.1.53

[phy213174-bib-0021] Varlamov, O. , C. L. Bethea , and C. T. Jr Roberts . 2015 Sex‐specific differences in lipid and glucose metabolism. Front Endocrinol (Lausanne). 5:241. doi:10.3389/fendo.2014.00241. 25646091 10.3389/fendo.2014.00241 PMC4298229

